# Provider readiness and implementation barriers for lung cancer screening in a safety-net system

**DOI:** 10.1016/j.pmedr.2025.103164

**Published:** 2025-07-03

**Authors:** Maria C. Mejia, Yu-Heng Hilary Ma, Lisa M. Lowenstein, Kiara K. Spooner, Gabrielle Duhon, Elisa E. Douglas, Viola Leal, Robert J. Volk

**Affiliations:** a Department of Population Health, Schmidt College of Medicine, Florida Atlantic University, Boca Raton, FL, USA; b Department of General Medical Oncology, The University of Texas MD Anderson Cancer Center, Houston, USA; c Department of Health Services Research, The University of Texas MD Anderson Cancer Center, Houston, TX, USA; d University of Houston, Population Health, 4349 Martin Luther King Blvd, Houston, TX 77204-2103, United States

**Keywords:** Lung cancer screening, Shared decision-making, Primary health care, Safety-net healthcare setting, Health disparities

## Abstract

**Objective:**

Lung cancer screening (LCS) with low-dose computed tomography is a critical preventive strategy, yet implementation remains complex, particularly in safety-net settings. This study assessed provider knowledge, attitudes, and readiness to implement LCS, including shared decision-making (SDM) and smoking cessation, within a large urban safety-net healthcare system.

**Methods:**

A cross-sectional survey was distributed between February and September 2023 to 200 healthcare providers, including physicians, nurses, advanced practice providers, trainees, and administrators in Houston, Texas. The 26-item instrument assessed familiarity with Centers for Medicare & Medicaid Services (CMS) LCS coverage, SDM billing, role preferences in LCS implementation, support needs, and readiness to engage in related activities.

**Results:**

Seventy-four providers responded (37 % response rate), and 60 completed the survey. Most were physicians (63 %), with specialties in family (45 %) and pulmonary medicine (20 %). While 77 % were familiar with CMS coverage, only 50 % were aware of SDM visit reimbursement, and 15 % knew the billing codes. Most identified physicians as key to SDM (96.7 %) and smoking cessation (93.3 %). Providers prioritized support in eligibility guidance (67 %), insurance coverage (60 %), decision aids (58 %), and cessation training (42 %). Preferred decision aids included exam room videos (70 %), waiting room videos (65 %), brochures (62 %), and electronic record-integrated tools (50 %). Most believed LCS benefits outweighed risks (92 %) and reported high readiness to engage in screening.

**Conclusions:**

Providers in safety-net settings support LCS implementation but report critical gaps in knowledge and infrastructure. Addressing these barriers through multidisciplinary collaboration, provider training, and improved decision support can enhance equitable delivery of lung cancer screening.

## 1 Introduction

Lung cancer remains the leading cause of cancer-related mortality worldwide, surpassing the combined deaths from breast, prostate, and colorectal cancers (Siegel et al., 2023). Early detection through low-dose computed tomography (LDCT) lung cancer screening (LCS) significantly reduces mortality by identifying cancer at earlier, more treatable stages (National Lung Screening Trial Research Team, 2011). et despite its clinical efficacy, lung cancer screening (LCS) uptake remains low (Mejia et al., 2022) with only 16.4 % of eligible adults in the U.S. screened in 2022 (Jemal and Fedewa, 2017). Barriers are particularly acute in safety-net health systems, which care for medically underserved populations and must navigate provider shortages, competing priorities, and disparities in patient access and health literacy (Mejia et al., 2022; Henderson et al., 2019).

Shared decision-making (SDM) is required by the Centers for Medicare & Medicaid Services (CMS) for LCS reimbursement and must include discussions about risks, benefits, eligibility, and tobacco cessation (Centers for Medicare and Medicaid Services (CMS), 2022). SDM ensures that patients make informed choices aligned with their values and preferences (Elwyn et al., 2012). However, many providers lack the time, training, and tools to conduct effective SDM in practice (Abubaker-Sharif et al., 2022; Lowenstein et al., 2018). Guidelines from the US Preventive Services Task Force (USPSTF) recommend annual LDCT screening for adults aged 50–80 with a 20 pack-year smoking history who currently smoke or quit within the past 15 years (US Preventive Services Task Force, 2021). The expansion of eligibility criteria offers an opportunity to reach more individuals at risk for lung cancer, though it may also add layers of complexity to implementation efforts within resource-limited healthcare systems. This study examines provider familiarity with LCS guidelines and CMS policies, attitudes toward shared decision-making, perceived malpractice risk, and readiness to implement LCS in a large safety-net health system. Our findings aim to inform strategies to expand screening access in high-need populations.

## 2 Methods

We conducted a cross-sectional survey between February and September 2023 among healthcare providers working in 18 community health centers affiliated with a large safety-net system in Houston, Texas. Eligible participants included physicians, advanced practice providers (APPs), nurses, trainees, and administrators providing care to potentially eligible LCS patients. Invitations were emailed to 200 providers with a link or Quick Response code to a REDCap (Harris et al., 2009) survey. Three reminder emails were sent to maximize response rates. The study was approved by the Institutional Review Board of The University of Texas MD Anderson Cancer Center. Participation was voluntary, and no incentives were provided.

The 26-item survey was adapted from previously validated instruments assessing readiness for LCS implementation (Volk and Foxhall, 2015; Lopez-Olivo et al., 2021) and constructs from the Organizational Readiness for Implementing Change (ORIC) framework (Shea et al., 2014) (see online appendix for the full instrument). Content areas included provider familiarity with CMS and professional guidelines for LCS, awareness of CMS coverage for shared decision-making SDM visits, preferences for roles in smoking cessation and SDM, perceived support needs for LCS implementation, and readiness to engage in key LCS-related activities. The survey underwent pilot testing and iterative refinement. Data were analyzed descriptively, with frequencies reported for categorical items and means and standard deviations calculated for ORIC scale items. Analyses were conducted using IBM SPSS Statistics, Version 29.0 (IBM Corp., Armonk, NY).

## 3 Results

Of the 200 providers invited, 74 responded (37 % response rate), and 60 completed the full survey (30 % completion rate). The majority were physicians (63.3 %, *n* = 38), followed by trainees (13.3 %, *n* = 8), nurses (11.7 %, *n* = 7), APPs (6.7 %, *n* = 4), and administrators (1.7 %, *n* = 1). Most respondents reported specialties in family medicine (45 %, *n* = 27), pulmonary medicine (20 %, *n* = 12), oncology (18.3 %, *n* = 11), and internal medicine (6.7 %, n = 4).

### 3.1 Knowledge and familiarity with LCS guidelines and CMS coverage

Only 31.7 % of respondents reported being very familiar with CMS coverage requirements for LCS, while 45.0 % were somewhat familiar and 23.3 % were not familiar. Although 50.0 % were aware that CMS covers SDM visits, 35.0 % were unaware and 15.0 % were unsure. Awareness of the associated CMS billing codes was even lower, only 15.0 % of providers were familiar with the codes, while 73.3 % were not aware and 11.7 % were unsure, despite required CMS documentation on billing for these services (Centers for Medicare and Medicaid Services (CMS), 2022; Elwyn et al., 2012). Familiarity with screening guidelines also varied. While 53.3 % of providers reported being very familiar with USPSTF recommendations, only 28.3 % reported the same level of familiarity with guidelines from the American Cancer Society and the American Academy of Family Physicians. These findings highlight gaps in provider knowledge and suggest fragmented dissemination of screening recommendations across professional organizations.

### 3.2 Preferences for who should implement LCS

**Fig. 1:**
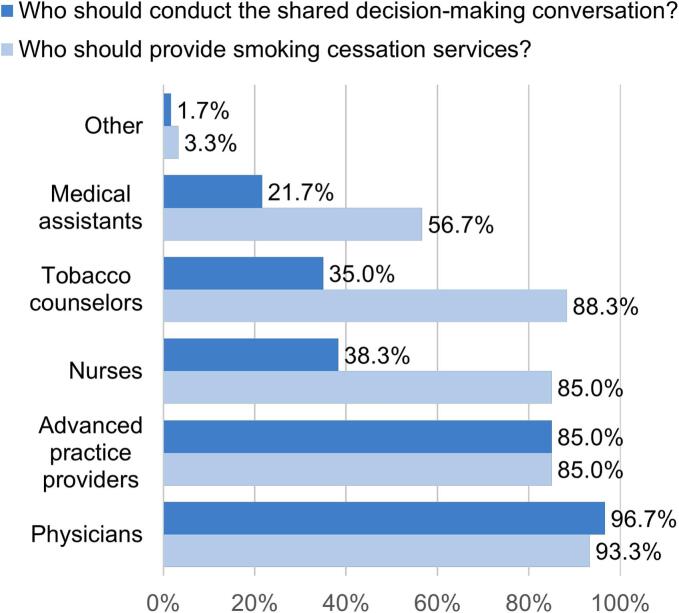
Preferred roles of healthcare team members in delivering shared decision-making conversations and smoking cessation counseling for lung cancer screening among providers in a safety-net health system, Houston, Texas, 2023.

Respondents favored multidisciplinary involvement in implementing LCS (Fig. 1). Providers overwhelmingly identified physicians as the lead clinicians for SDM (96.7 %, *n* = 58) and smoking cessation counseling (93.3 %, *n* = 56). However, APPs (85.0 %, *n* = 51), nurses (38.3 %, *n* = 23), and tobacco counselors (35.0 %, *n* = 21) were also seen as key contributors for SDM. These findings reinforce calls for a team-based, interdisciplinary model to support screening delivery.

### 3.3 Support needs

The most commonly cited needs were clarity on eligibility (66.7 %, *n* = 40), insurance coverage (60.0 %, *n* = 36), and access to patient decision aids (58.3 %, *n* = 35). Training in SDM (45.0 %, *n* = 27) and smoking cessation counseling (41.7 %, *n* = 25) was also identified as a critical gap. Guidance on referring patients for LDCT (58.3 %, n = 35) and clarity on follow-up for abnormal findings (50.0 %, *n* = 30) were also significant needs. These responses are consistent with prior findings that many providers feel underprepared to implement LCS within routine workflows.

### 3.4 Decision aids preferences

The most favored decision aid formats were videos shown in the exam room (70.0 %, *n* = 42) or waiting room (65.0 %, *n* = 39), brochures for home use (61.7 %, *n* = 37), MyChart-delivered tools (50.0 %, *n* = 30), and printable summaries for office visits (46.7 %, *n* = 28). These preferences align with recent research highlighting the effectiveness of multimodal patient education in cancer screening.

### 3.5 Perceptions of lung cancer screening benefits and risks

Most providers viewed LCS positively, with 91.7 % agreeing or strongly agreeing that its benefits outweigh the risks (Table 1). Nearly all respondents (93.3 %) disagreed with the notion that patient education about LCS is unnecessary, highlighting broad support for informed patient engagement. Perceptions of malpractice liability were mixed. One-third of respondents (33.4 %) believed that not offering LCS could expose them to legal risk, while 40.0 % were neutral and 25.0 % disagreed. Additionally, 88.1 % disagreed or strongly disagreed with the idea that LCS causes more harm than good, reflecting a strong belief in its clinical value. On resource prioritization, 61.7 % disagreed that efforts should focus solely on smoking prevention over screening, though 25.0 % were neutral suggesting some uncertainty about balancing prevention and early detection strategies.

**Table 1:** Characteristics, perceptions, and readiness to implement lung cancer screening among healthcare providers in a safety-net health system, Houston, Texas, 2023 (*N* = 74).

Perceptions of Lung Cancer Screening Benefits and Risks		
Agree or Strongly Agree that	n	Percent
The benefits of lung cancer screening outweigh the risks	55	91.7
Not ordering lung cancer screening puts a physician at risk for malpractice liability	20	33.3
I do not have time to discuss lung cancer screening with my patients	12	20.0
Health care resources should be directed toward preventing smoking rather than screening for lung cancer	8	13.3
There is no need to educate patients about lung cancer screening because they already want to be screened.	2	3.3
Lung cancer screening in real world clinical practice will lead to more harms than benefits for patients	2	3.3
*Readiness to Implement Lung Cancer Screening*		
Agree or Strongly Agree that	**n**	**Percent**
Refer patients to a lung cancer screening program	55	91.7
Identify patients eligible for lung cancer screening	53	88.3
Engage patients in informed/shared decision making about lung cancer screening prior to referral	53	88.3
Follow up with patients who are screened and have an abnormal finding	52	86.7
Use a patient decision aid about lung cancer screening	49	81.7
Provide tobacco treatment services to patients who smoke	48	80.0
Manage their other health problems of patients diagnosed with lung cancer during cancer treatment	45	75.0

### 3.6 Readiness to implement lung cancer screening

Overall, providers reported high readiness to implement LCS (Table 1). Most felt prepared to identify eligible patients (88.3 %) and engage in shared decision-making (88.4 %). A majority also expressed readiness to use decision aids (81.6 %), refer patients to screening programs (94.8 %), and follow up on abnormal results (86.6 %). Regarding related care, 80.0 % of providers felt prepared to offer tobacco treatment services, and 75.0 % felt confident managing other health issues during lung cancer treatment. While 61.7 % disagreed that they lacked time to discuss LCS, 20.0 % of respondents reported time constraints, indicating that workload remains a barrier for a subset of providers.

## 4 Discussion

Despite the well-established benefits of low-dose computed tomography in reducing lung cancer mortality, uptake remains low, with only 16.4 % of eligible adults screened in 2022 (Jemal and Fedewa, 2017). This is particularly concerning in safety-net healthcare systems, where medically underserved populations face multiple barriers, including social determinants of health, limited resources, and competing clinical demands (Mejia et al., 2022; Henderson et al., 2019; Carter-Harris et al., 2020). Given the significant burden of tobacco-related morbidity in these communities, improving LCS uptake is both a clinical priority and public health imperative.

This study provides important insights into provider readiness, knowledge gaps, and implementation barriers within a large urban safety-net system. Most providers recognized the value of LCS, yet significant gaps in knowledge about CMS coverage policies, billing codes, and other professional organizations guidelines were identified. Notably, only 15 % were familiar with CMS billing codes for SDM visits, a prerequisite for reimbursement (Centers for Medicare and Medicaid Services (CMS), 2022; Elwyn et al., 2012). This gap may hinder both implementation and sustainability, particularly in resource-constrained settings where missed reimbursements can strain operational capacity.

Providers broadly endorsed multidisciplinary involvement in LCS, with strong support for team-based models that include advanced practice providers, nurses, and tobacco counselors (Abubaker-Sharif et al., 2022; Lowenstein et al., 2018). Task-sharing and care coordination models are vital for embedding LCS into routine preventive care, especially in primary care settings managing complex patients with limited time (Volk and Foxhall, 2015; Khatri et al., 2023).

Nearly all respondents (96.7 %) identified physicians as pivotal in conducting SDM, indirectly affirming their support for CMS requirements. Although explicit attitudes toward the SDM mandate were not directly measured, high reported readiness (88.4 %) to engage in SDM, alongside the overwhelmingly positive perception of LCS benefits (91.7 %), suggest providers are supportive of integrating SDM into clinical practice.

Providers identified several critical support needs, notably clearer eligibility guidelines, better information on insurance coverage, and improved access to patient decision aids. They expressed preferences for multimodal educational materials, including videos for exam and waiting rooms, electronic health record-integrated tools, and patient-accessible formats like MyChart resources. These diverse formats are especially valuable in safety-net settings where linguistic, literacy, and cultural factors may pose barriers to patient understanding and engagement.

Concerns regarding potential malpractice liability also emerged, with one-third of respondents worried about legal risks associated with no offering LCS, while many others were uncertain (Farjah et al., 2021). These mixed views highlight the need for institutional policies and professional training that clarify clinical and legal standards, particularly in preventive care.

Time constraints emerged as another common barrier. Although most respondents reported having time to discuss LCS, 20 % indicated they did not, consistent with prior studies noting that SDM is particularly complex for patients with multiple chronic conditions or psychosocial risk factors (Kale et al., 2024). Embedding SDM into routine workflows using tools such as electronic health record prompts, pre-visit planning, or nurse-led counseling may help alleviate this burden. Consistent with previous qualitative research, our study highlights that time limitations and inadequate decision support tools hinder effective SDM, potentially leading to gaps in patient understanding and engagement (Wiener et al., 2018; Lewis et al., 2023).

Despite these challenges, providers demonstrated strong readiness to implement LCS activities, including patient identification, SDM, and follow-up management. This readiness assessed using the Organizational Readiness for Implementing Change (ORIC) framework (Shea et al., 2014), highlights an opportunity to develop sustainable, patient-centered programs. It further underscores provider readiness as a crucial, yet often overlooked, determinant of successful screening program implementation. Our findings align with national studies, indicating that healthcare organizations with higher readiness and commitment to change have greater LCS utilization (Lewis et al., 2023). Targeted strategies aimed at enhancing clinician and staff preparedness could significantly improve LCS rates (Lewis et al., 2023).

Ultimately, while patient engagement and structural access remain essential, empowering providers with targeted training, accessible tools, and systemic supports can bridge existing gaps between guidelines and clinical practice. Strategic investments in provider-centered resources and multidisciplinary collaboration can significantly enhance LCS uptake, advancing health equity in underserved populations.

### 4.1 Strengths and limitations

This study provides timely, actionable insights into provider readiness within a significant urban safety-net healthcare setting. However, generalizability may be limited by the single-site design. The moderate response rate (37 %) and survey completion rate (30 %) introduce potential response bias, possibly favoring providers already supportive of LCS. The low percentage reporting time constraints (20 %) may reflect social desirability bias, leading to underreporting of challenges faced in clinical practice. Additionally, subgroup analyses were limited due to small sample sizes. Detailed demographic or specialty-specific data were not available for non-responders due to the anonymous nature of the survey. However, all invited participants were from the same safety-net health system and affiliated medical schools, which share a similar provider composition to those who responded. Nonetheless, the findings effectively highlight critical provider perspectives often overlooked in research on LCS implementation.

## 5 Conclusion

Lung cancer screening represents a crucial opportunity to reduce mortality, particularly among underserved populations disproportionately affected by lung cancer morbidity and mortality. Our findings indicate that providers within safety-net systems are motivated and generally ready to implement LCS but face significant barriers related to knowledge gaps, time limitations, and insufficient infrastructure.

Addressing these challenges requires targeted provider training, robust decision-support tools, clear reimbursement pathways, and workflow optimizations. Equally critical is a systems-level commitment to multidisciplinary collaboration, ensuring equitable access and patient engagement across diverse populations. As LCS eligibility criteria expand, prioritizing resources and tailored interventions in safety-net environments is essential to prevent widening disparities.

Ultimately, strengthening LCS implementation within safety-net settings will advance health equity, delivering high-value, evidence-based cancer prevention effectively and equitably to all at-risk communities.

## CRediT authorship contribution statement

**Maria C. Mejia:** Writing – review & editing, Writing – original draft, Visualization, Methodology, Investigation, Data curation, Conceptualization. **Yu-Heng Hilary Ma:** Writing – review & editing, Methodology, Investigation, Conceptualization. **Lisa M. Lowenstein:** Writing – review & editing, Resources, Methodology, Investigation, Conceptualization. **Kiara K. Spooner:** Writing – review & editing, Methodology, Investigation. **Gabrielle Duhon:** Visualization, Project administration, Methodology, Investigation, Data curation. **Elisa E. Douglas:** Resources, Methodology, Investigation, Conceptualization. **Viola Leal:** Writing – review & editing, Visualization, Investigation, Data curation. **Robert J. Volk:** Writing – review & editing, Writing – original draft, Visualization, Validation, Supervision, Project administration, Methodology, Investigation, Funding acquisition, Formal analysis, Data curation, Conceptualization.

## Funding

This work was supported by a grant from The University of Texas MD Anderson Cancer Center Community Outreach and Engagement Fund for Underserved Texans, by annual distributions of the Permanent Health Fund endowment received by The University of Texas MD Anderson Cancer Center from the state legislature, by a grant from NIH/NCI under award number P30CA016672 and used the Decision Science Core and Clinical Protocol and Data Management, and in part by a grant from the 10.13039/100004917Cancer Prevention and Research Institute of Texas (RP160674). The funding sources had no involvement in the study design, data collection, analysis or interpretation, writing of the manuscript, or the decision to submit the article for publication.

## Declaration of competing interest

The authors declare that they have no known competing financial interests or personal relationships that could have appeared to influence the work reported in this paper.

## Appendix A Supplementary data

This appendix includes the full 26-item survey instrument administered to healthcare providers. The survey was designed to assess provider familiarity with lung cancer screening (LCS) guidelines and policies, attitudes toward shared decision-making (SDM) and smoking cessation, perceived support needs, and readiness to implement LCS within a safety-net healthcare system. The instrument incorporates items adapted from previously validated measures of implementation readiness and the Organizational Readiness for Implementing Change (ORIC) framework. Image 1
