# Supplementary material for: Provider readiness and implementation barriers for lung cancer screening in a safety-net system
Source: Prev Med Rep. 2025 Jul 3;56:103164. doi: 10.1016/j.pmedr.2025.103164 (PMC12272585; doi:10.1016/j.pmedr.2025.103164)
Supplement: Supplementary file 1 — This appendix includes the full 26-item survey instrument administered to healthcare providers. The survey was designed to assess provider familiarity with lung cancer screening (LCS) guidelines and policies, attitudes toward shared decision-making (SDM) and smoking cessation, perceived support needs, and readiness to implement LCS within a safety-net healthcare system. The instrument incorporates items adapted from previously validated measures of implementation readiness and the Organizational Readiness for Implementing Change (ORIC) framework. [file mmc1.pdf]

Study Description and Consent

The goal of this research study is to better understand lung cancer screening within your healthcare institution and improve educational tools for patients considering screening.

If you do not wish to participate, you may close this page. If you agree to participate in the study, you will complete a survey about your practice, general information about lung cancer screening, tobacco cessation, and so on. No health information about you will be collected. This survey will take about 5-10 minutes to complete.

Researchers may collect names and email address, but this information will be confidential. You may be contacted in the future to participate in other activities related to this study, but you are free to refuse any further participation. Data collected in this survey will be aggregated and shared with the study team members from UTHealth, Baylor College of Medicine, and MD Anderson.

There is no cost to you for taking part in this study. There are no benefits for you in this study. Your participation is completely voluntary. You may choose not to take part in this study.

Up to 340 participants total will be enrolled in this study. Up to 200 participants from Harris Health System, Baylor College of Medicine, MD Anderson, and UTHealth will be enrolled in this part of the study.

As an employee, your participation or non-participation in this study will not affect your employment status at the institution or your employee benefits. You will not be impacted (favorably or unfavorably) based on your study participation decision. None of the following will be affected:

- Performance evaluations
- Career advancement
- Assignments
- Time off approvals

Consent Statement

You have read the description of the study, and have decided to participate in the research project described here. You understand that you may refuse to answer any (or all) of the questions at this or any other time. You understand that there is a possibility that you might be contacted in the future about this, but that you are free to refuse any further participation if you wish.

You may withdraw your authorization at any time, in writing, for any reason as long as that information can be connected to you. You can learn more about how to withdraw your authorization by calling 713-792-6477 or by contacting the study chair (Dr. Robert Volk) at 713-563-0020.

Provider Survey

Researchers at Baylor College of Medicine, UTHealth, and MD Anderson are interested in the views of providers and other health professionals who provide care to patients in the Harris Health System about implementing lung cancer screening. This anonymous survey will provide important information about readiness and potential challenges providers and other health professionals may face as they consider lung cancer screening for their patients. We appreciate your participation.

What is your primary role in your clinic?

- ◯ Physician
- ◯ Nurse
- ◯ APP
- ◯ Administrator
- ◯ Trainee (including fellows, residents, and students)
- ◯ Other

Other: ______________________________

If you are a health care provider, what is your specialty training?

- ◯ Family Medicine
- ◯ Internal Medicine
- ◯ Oncology
- ◯ Pulmonary Medicine
- ◯ Other

Other: ______________________________

Please select your employer.

- ◯ MD Anderson Cancer Center
- ◯ UTHealth
- ◯ Baylor College of Medicine
- ◯ Harris Health System

What is your primary practice location? Check all that apply.

- ☐ Acres Home Health Center
- ☐ Aldine Health Center
- ☐ Baytown Health Center
- ☐ Casa De Amigos Health Center
- ☐ Cypress Health Center
- ☐ Danny Jackson Health Center
- ☐ El Franco Lee Health Center
- ☐ Gulfgate Health Center
- ☐ Martin Luther King Jr. Health Center
- ☐ Northwest Health Center
- ☐ Settegast Health Center
- ☐ Squatty Lyons Health Center
- ☐ Strawberry Health Center
- ☐ Thomas Street Health Center
- ☐ Vallbona Health Center
- ☐ Lyndon B. Johnson Hospital
- ☐ Ben Taub Hospital
- ☐ Smith Clinic
- ☐ Other

Other: ______________________________

How familiar are you with CMS coverage requirements for lung cancer screening (LCS) with a low-dose CT scan (LDCT)?

- ◯ Very familiar
- ◯ Somewhat familiar
- ◯ Not familiar

Are you aware that CMS covers a patient counseling and shared decision-making visit for lung cancer screening?

- ◯ Yes
- ◯ No
- ◯ I'm not sure

Are you aware of the CMS billing code for shared-decision making visit for lung cancer screening?

- ◯ Yes
- ◯ No
- ◯ I'm not sure

How familiar are you with clinical guidelines about lung cancer screening from the following organizations?

	Very familiar	Somewhat familiar	Not familiar
United States Preventive Services Task Force	◯	◯	◯
American Cancer Society	◯	◯	◯
American Academy of Family Physicians	◯	◯	◯

The following questions are about implementing lung cancer screening in your clinical practice setting.

Who should provide smoking cessation interventions to your patients? Check all that apply.

- ☐ Physicians
- ☐ Nurses
- ☐ Tobacco counselors
- ☐ APPs (i.e., nurse practitioners, physician assistants)
- ☐ Medical assistants
- ☐ Other

Other: __________________________________

Who should conduct the shared decision-making conversation about lung cancer screening with your patients? Check all that apply.

- ☐ Physicians
- ☐ Nurses
- ☐ Tobacco counselors
- ☐ APPs (i.e., nurse practitioners, physician assistants)
- ☐ Medical assistants
- ☐ Other

Other: __________________________________

What support would be most helpful to you in implementing lung cancer screening? Check all that apply.

- ☐ Clarity on lung cancer screening eligibility guidelines
- ☐ Clarity on insurance/CMS coverage
- ☐ Patient decision aids/educational tools
- ☐ Skills training in shared decision making
- ☐ Guidance on where to refer patients for LCDT screening
- ☐ Training in smoking cessation interventions
- ☐ Clarity on follow up for abnormal findings
- ☐ Other

Other: __________________________________

What kinds of decision aids about lung cancer screening would be most helpful to your patients? Check all that apply.

- ☐ Video patients can watch in the exam room
- ☐ Video patients can watch in the waiting room
- ☐ A brochure patients can take home
- ☐ A summary to review with patients during an office visit
- ☐ A decision aid sent to patients via MyChart
- ☐ Other

Other: ________________________________

What are your opinions about lung cancer screening with a low-dose CT scan for patients who smoke and are at high-risk for lung cancer? Indicate how much you agree or disagree with each statement.

	Strongly Disagree	Disagree	Neutral	Agree	Strongly Agree
The benefits of lung cancer screening outweigh the risks.	○	○	○	○	○
There is no need to educate patients about lung cancer screening because they already want to be screened.	○	○	○	○	○
Not ordering lung cancer screening puts a physician at risk for malpractice liability.	○	○	○	○	○
I do not have time to discuss lung cancer screening with my patients.	○	○	○	○	○
Health care resources should be directed toward preventing smoking rather than screening for lung cancer.	○	○	○	○	○
Lung cancer screening in "real world" clinical practice will lead to more harms than benefits for patients.	○	○	○	○	○

Characteristics of lung cancer screening programs in primary care settings are listed below. Indicate how ready you are to implement each activity.

	Strongly Disagree	Disagree	Neither	Agree	Strongly Agree
I am ready to identify patients eligible for lung cancer screening.	○	○	○	○	○

I am ready to engage patients in informed/shared decision making about lung cancer screening prior to referral.	○	○	○	○	○
I am ready to use a patient decision aid about lung cancer screening.	○	○	○	○	○
I am ready to refer patients to a lung cancer screening program.	○	○	○	○	○
I am ready to follow up with patients who are screened and have an abnormal finding.	○	○	○	○	○
For patients who are diagnosed with lung cancer, I am ready to manage their other health problems during cancer treatment.	○	○	○	○	○
I am ready to provide tobacco treatment services to patients who smoke.	○	○	○	○	○
